# Serum PLA2R-IgG4/PLA2R-IgG ratio dynamics reveal pathogenic autoantibody subclass switch during progression of PLA2R-associated membranous nephropathy

**DOI:** 10.1080/07853890.2025.2610874

**Published:** 2026-01-05

**Authors:** Yongzhong Zhong, Yunyun Liu, Dan Zhou, Jing Tian, Dacheng Chen, Dandan Liang, Shaoshan Liang, Tianyu Zhen, Xiaodong Zhu, Biao Huang, Caihong Zeng

**Affiliations:** ^a^ National Clinical Research Center for Kidney Diseases, Jinling Hospital, Affiliated Hospital of Medical School, Nanjing University, Nanjing, China; ^b^ Department of Nephrology, Hangzhou TCM Hospital Affiliated to Zhejiang Chinese Medical University, Hangzhou, China; ^c^ Jinling Clinical Medical College, Nanjing University of Chinese Medicine, Nanjing, China; ^d^ College of Life Sciences and Medicine, Zhejiang Sci-Tech University, Hangzhou, China

**Keywords:** Subclass switch, anti-PLA2R antibody, membranous nephropathy

## Abstract

**Background:**

Pathogenic autoantibody subclass switch has been found in lots of autoimmune disease. However, the information on anti-phospholipase A2 receptor antibody subclass switch in membranous nephropathy (MN) is limited and controversial. Here, we aim to uncover the subclass change during the PLA2R-associated MN progression.

**Methods:**

Biopsy-proven PLA2R-associated MN cases with sufficient tissue for light microscopy, immunofluorescence, and electron microscopy (October 2022 - March 2023) were included. Serum levels of PLA2R-IgG4 and PLA2R-IgG were measured by TRFIA. The correlation of the ratio with EM stage and other clinical parameters was analyzed.

**Results:**

Among 116 enrolled patients, glomerular IgG1 (*r* = 0.15, *p* = .01; *r* = 0.18, *p* = .002) and IgG3 (*r* = 0.17, *p* = .005; *r* = 0.27, *p* < .001) intensities were positively correlated with C3 and C1q intensities, respectively. The PLA2R-IgG4/PLA2R-IgG ratio was significantly positively correlated with serum albumin (*r* = 0.26, *p* = .005) but inversely correlated with both the intensity of glomerular IgG1 (*r* = −0.20, *p* = .03) and IgG3 deposits (*r* = −0.24, *p* = .009), as well as with C1q staining intensity (*r* = −0.27, *p* = .004). The median PLA2R-IgG4/PLA2R-IgG ratio significantly increased with pathological stage (Stage I: 18.92%; Stage II: 39.74%; Stage III: 59.38%; Stage IV: 68.99%) and was strongly positively correlated with EM stage (*r* = 0.52, *p* < .001). Advanced EM stages were observed more frequently with higher PLA2R-IgG4/PLA2R-IgG ratio.

**Conclusions:**

During the disease progression, EM stages were correlated with altered autoantibody IgG subclass profiles: early stages featured IgG1 or IgG3 autoantibodies, while late EM stages shifted to IgG4 predominance.

## Key Messages

- PLA2R-IgG4/PLA2R-IgG ratio raised with pathological stage progress in PLA2R associated MN.
- Lower PLA2R-IgG4/PLA2R-IgG was associated with Higher PLA2R-IgG levels and lower serum albumin, which indicated disease activity and severity.
- During the disease progress, not only the level of total anti-PLA2R antibody fluctuate, but the proportions of its IgG subclass also exhibit dynamic change.

## Introduction

Primary membranous nephropathy (PMN) is the most common cause leading to nephrotic syndrome in non-diabetic adults, and nearly one-third patients progressed to end stage kidney disease. This renal-limited autoimmune disease is caused by circulating autoantibodies that target glomerular podocyte antigens, which phospholipase A2 receptor (PLA2R) account for 60–80% [1]. The discovery of anti-PLA2R antibody has revolutionary change the management of the PMN, since serum anti-PLA2R antibody have been found to be highly specific diagnostic and prognostic biomarkers [2]. Among the four subclasses of IgG deposits in the glomeruli, IgG4 is the predominant within glomerular immune complex, followed by IgG1. Other subclasses may be detectable occasionally but typically exhibit lower staining intensities [3]. Circulating anti-PLA2R antibody predominantly but not exclusively belong to the IgG4 subclass, lower levels of IgG1 or IgG3 subclass autoantibodies are detectable in some patients’ serum [4,5]. Each IgG subclass has a different biological function, such as the binding affinity of C1q and the ability of activation the complement pathway. Thus, the subclass distribution of autoantibody might be associated with the severity and the progression of autoimmune disease. Subclass switch of pathogenic autoantibody is found in some autoimmune diseases during the disease progression [6–12]. These studies revealed that the IgG subclass critically determine disease progression and therapeutic responses.

Recent studies utilizing immunostaining approaches have delineated glomerular IgG subclass redistribution patterns deposited in glomeruli during histopathological progression [13,14]. However, findings regarding subclass dynamics remain divergent, with available data still limited. While one study have documented a temporal IgG subclass switch – characterized by progressive predominance of IgG4 over IgG1 during chronic antigenic exposure [13]. Notably, no subclass switch from IgG1 to IgG4 were found in other study, which challenging the conventional switching paradigm [14]. This subclass stability appears reinforced by post-transplantation protocol biopsy surveillance data, where recurrent MN lesions IgG4 maintained predominant subclass [15]. However, the above studies focus on the IgG subclass deposited in the glomeruli. The autoantibodies deposited in the glomeruli are only small partial of the total antibody, which most of the autoantibodies are in circulation when the disease is activity. To our knowledge, the quantitative evolution of serum IgG4 subtype anti-PLA2R antibodies (PLA2R-IgG4) within total anti-PLA2R antibodies (PLA2R-IgG) across distinct disease stages remains unelucidated.

In this study, we aimed to assess the relationship between PLA2R-IgG4/PLA2R-IgG and Ehrenreich-Churg electron microscopy (EM) stages, and attempt to explored the predominant IgG autoantibody subclass in different EM stages. Furthermore, we sought correlations between clinical parameters and PLA2R-IgG4/PLA2R-IgG to enhance the understanding of the pathogenesis of PMN.

## Patients and methods

### Patient selection

Patients with native kidney biopsy-proven MN in National Clinical Research Center for Kidney Diseases from October 2022 to May 2023 were carefully screened. The including criteria were as follows: 1. Age more than 18 years old; 2. The diagnosis of MN was confirmed by kidney biopsy; 3. The PLA2R staining were positive in the tissue section and exhibited serum PLA2R ELISA results more than 2 RU/ml; 4. Biopsies with glomeruli available for light microscopy, immunofluorescence and electron microscopy. The exclusion criteria were as follows:1. Potential secondary MN, such as tumor associated-MN, hepatitis B virus associated-MN, membranous lupus nephritis and so on; 2. Superimposed with another glomerulopathy lesions, such as IgA nephropathy, diabetic nephropathy, ANCA associated nephritis; 3. No glomeruli founded in the EM examination; 4. Not enough serum for the detection of anti-PLA2R antibody or the level of anti-PLA2R antibody were below the detection limitation; 5. Patients received immunosuppressive therapy before sampling. This study strictly adhered to the ethical principles of the Declaration of Helsinki, with the study protocol reviewed and approved by the Medical Ethics Committee of the Jinling Hospital (2022DZKY-053-01), and written informed consent was obtained from all participants.

### Clinical data collection

The demographics and clinical parameters of patients at the time of renal biopsy were collected such as gender, age, the duration from disease onset to kidney biopsy, and the presence of hypertension or diabetes. Additionally, laboratory parameters, including albumin (Alb), creatinine (Cr), estimated-glomerular filtration rate (eGFR) (calculated by Chronic Kidney Disease Epidemiology Collaboration (CKD-EPI) formula) and 24-h urinary protein excretion, were meticulously recorded.

### Pathological examination and procedure and definition

Pathological assessment encompassed a comprehensive examination through light microscopy (LM), immunofluorescence (IF), and electron microscopy (EM). Tissue for light microscopy was routinely fixed in formalin and embedded in paraffin. The paraffin sections were stained routinely with hematoxylin and eosin, periodic acid-Schiff, periodic acid-silver methenamine, and Masson. Acute tubulointerstitial injury index (ATI) including active, potentially reversible damage, such as the tubular epithelial cell degeneration or necrosis, tubular dilatation, and intraluminal casts or the interstitial edema. Chronic tubulointerstitial injury index (CTI) represented irreversible, chronic scarring, such as tubular atrophy and interstitial fibrosis. Based on the percentage of cortical area affected, the score was graded on a scale of 0–3 (0: none or less than 10%; 1: 10–25%; 2: 26–50%; 3: >50% involvement). This scoring system aligns with the criteria established in prior studies [16].

Direct immunofluorescence was performed on frozen sections with the following antibody panels: IgG/IgA/IgM/C3/C1q/kappa/lambda-light chains (Kent Laboratories) and IgG1/IgG2/IgG3/IgG4 (The binding site). Indirect immunofluorescence was utilized to detect glomerular PLA2R antigen on paraffin-embedded tissue sections. The procedure involved dewaxing, rehydration, blocking, and subsequent incubation with rabbit polyclonal anti-human PLA2R antibody (Sigma-Aldrich) followed by FITC-conjugated pig anti-rabbit IgG antibody (Dako). Immunofluorescence staining intensity was semi-quantitatively scored during the routine biopsy evaluation from 0 to 3+ (0, negative; 1+, weak staining; 2+, moderate staining; 3+, strong staining).

Transmission electron microscopy was performed using ultrathin sections embedded in Eponate 12 resin and stained with lead citrate and uranium acetate to observe the ultrastructure. According to the Ehrenreich–Churg classification system, MN was classified into four stages [17]. Stage I: Small, discrete subepithelial electron-dense deposit, with no spike formation and GBM thickening. Stage II: Spike formation was diffuse with only a few deposits incorporated into the GBM, giving a ‘comb-like’ appearance; Stage III: Deposits are partially encircled by GBM matrix, forming a ‘halo’ around the deposits. Spikes merge, leading to thickened GBM with alternating electron-dense (deposits) and electron-lucent (new matrix) regions. Deposits may appear intramembranous; Stage IV: most immune complex deposits were absorbed and formed the lucent areas, the GBM were irregular thickened. This ultrastructural staging and immunofluorescence results was performed retrospectively on every biopsy by two independently renal pathologists (D.L. and S.L.) blinded to each other and the result of PLA2R-IgG4/PLA2R-IgG.

### Detection of PLA2R-IgG and PLA2R-IgG4

The serum of patients was collected before biopsy and stored at −80 °C before detecting the concentration of PLA2R-IgG and PLA2R-IgG4 using TRFIA methods. The testing methods has been published in our previous work [18,19]. The assay was performed as follows: 100 μL of diluted serum samples (1:200 for anti-PLA2R-IgG detection; 1:20 for anti-PLA2R-IgG4 detection) were aliquoted into rPLA2R-coated microtiter plates (5 μg/mL coating concentration). Following a 1-h incubation at 25 °C under continuous agitation (500 rpm), unbound components were removed through three consecutive wash cycles. Subsequently, 100 μL of detection antibodies were added: europium-conjugated goat anti-human IgG for total IgG detection or mouse anti-human IgG4 antibodies for IgG4-specific detection. After a secondary 1-h incubation at 25 °C with shaking, six rigorous wash cycles were performed to remove unbound conjugates. The detection phase commenced with addition of 200 μL enhancement solution per well, followed by 5-min orbital mixing to optimize signal development. Fluorescence measurements were conducted using the AutoDELFIA_1235_ system, which automatically calculated anti-PLA2R antibody concentrations through built-in calibration curves based on europium fluorescence intensity, and more detail methods could be seen in our previous study [18,19]. The positive cutoff for diagnosing PLA2R-associated MN was defined as 1990 ng/ml for PLA2R-IgG and 161.2 ng/ml for PLA2R-IgG4 according our previous results.

### Statistical analyses

For descriptive statistics, data are presented as mean ± standard deviation or median (interquartile range) when appropriate. The correlation between several parameters (nonparametric distributions) was analyzed by Spearman’s rank coefficient of correlation; Mann–Whitney *U*, and Kruskal–Wallis tests were used for comparison between groups according the data distribution pattern. Univariate analysis followed by multiple Logistic regression was used to determine independent predictors of EM stages. All tests were two-sided and *p* < .05 were considered statistically significant. Statistical analyses were performed by the SPSS statistical software package, version 20.0 (SPSS Inc., Chicago, IL), MedCalc (MedCalc Software Ltd) and GraphPad Prism 8.0 (GraphPad Software).

## Results

### Clinical characters of the patients enrolled in this study

**Figure 1.**
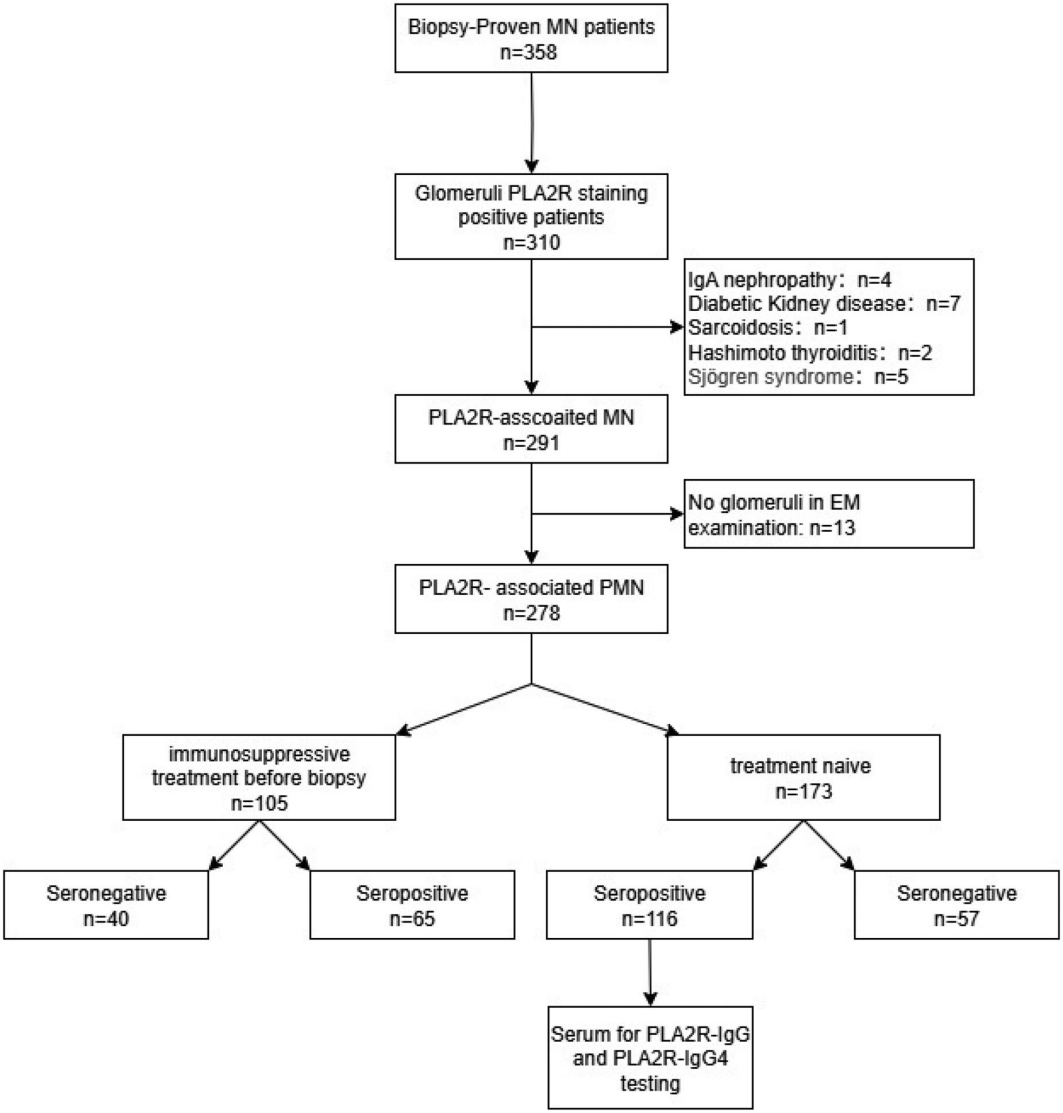
Flowchart of study participants.

A total of 116 patients were enrolled in this study, the enrolled scheduled were shown in Figure 1. The mean age at diagnosis was 50.04 ± 11.51 years old, and male account for 67% (*n* = 78). The median course was 4.0 months (1.0, 9.0). The median serum creatinine and eGFR was 0.82 mg/dl (0.69, 0.99) and 103.50 ml/min/1.73m^2^ (86.00, 114.00), respectively. The median 24-h proteinuria was 4.29 g/24 h (2.73, 8.16), and mean serum albumin was 26.86 ± 5.69 g/L. The proportion of nephrotic syndrome was 55.17% (*n* = 64). The median level of PLA2R-IgG and PLA2R-IgG4 was 11296.12 ng/ml and 4646.41 ng/ml, respectively. And the median PLA2R-IgG4/PLA2R-IgG ratio was 0.40 (0.28, 0.52). The detailed clinical parameters of these patients were shown in Table 1. The distribution of patients across EM stages was as follows: Stage I (*n* = 12), Stage II (*n* = 80), Stage III (*n* = 20), and Stage IV (*n* = 4). Due to the limited number of cases in Stage I (*n* = 12) and Stage IV (*n* = 4), patients were categorized into two groups for analysis: an early-stage group (Stages I and II) and an advanced-stage group (Stages III and IV). No significant differences in clinical presentations were observed between these two groups (Table 1).

**Table 1.** Demographic and clinical parameters of patients enrolled in this study.

Variables	Total (*n* = 116)	I + II (*n* = 92)	III+IV (*n* = 24)	*p*
Age, years	50.04 ± 11.51	50.86 ± 11.03	46.92 ± 12.99	0.14
Male, *n* (%)	78 (67.24)	61 (66.30)	17 (70.83)	0.67
HP, *n* (%)	45 (38.79)	35 (38.04)	10 (41.67)	0.75
DM, *n* (%)	13 (11.21)	11 (11.96)	2 (8.33)	0.89
Course, months	4.00 (1.00, 9.00)	3.00 (1.00, 8.00)	4.00 (1.75, 12.00)	0.50
Serum Albumin, g/l	26.86 ± 5.69	26.73 ± 5.57	27.39 ± 6.24	0.61
Serum Creatinine, mg/dl	0.82 (0.69, 0.99)	0.81 (0.69, 0.95)	0.89 (0.68, 1.02)	0.27
eGFR, ml/min/1.73m^2^	103.50 (86.0, 114.00)	104.00 (88.00, 114.25)	101.00 (78.00, 110.25)	0.30
Upro, g/24h	4.29 (2.73, 8.16)	4.40 (2.71, 7.99)	3.80 (2.73, 8.37)	0.99
NS, *n* (%)	64 (55.17)	52 (56.52)	12 (50.00)	0.57
PLA2R-IgG, ng/ml	11296.12 (4840.06, 30972.44)	14275.54 (5596.26, 32414.13)	8904.26 (3070.55, 15707.79)	0.09
PLA2R-IgG4, ng/ml	4646.41 (1702.12, 11185.79)	4355.52 (1702.12, 11711.95)	6128.82 (1827.99, 8669.57)	0.95
PLA2R-IgG4/PLA2R-IgG	0.40 (0.28, 0.52)	0.38 (0.26, 0.46)	0.60 (0.42, 0.68)	**<.01**

HP: hypertension; DM: diabetic mellites; Upro: 24-h urine protein; NS: nephrotic syndrome.

### Relationship between the intensity of IgG subclass and C3 and C1q deposited in renal biopsy

**Figure 2.**
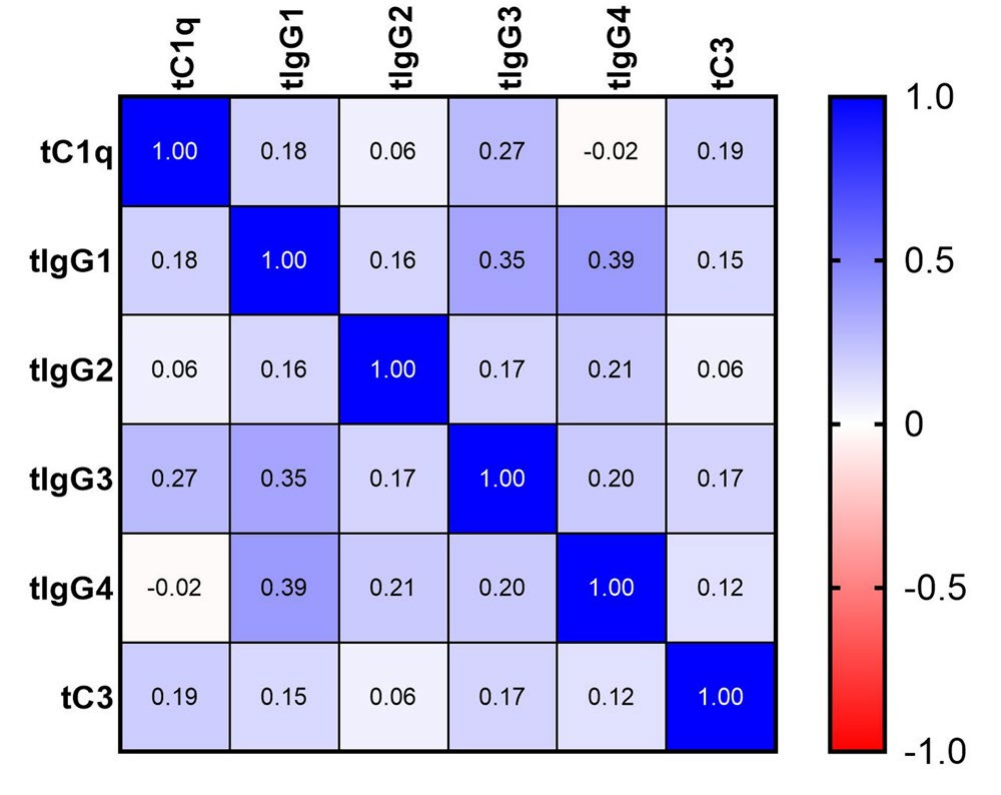
The correlation of the intensity of IgG subclass with the intensity of C3 and C1q deposited in glomeruli. tC1q: C1q deposited in the glomeruli; tC3: C3 deposited in the glomeruli; tIgG1: IgG1 deposited in the glomeruli; tIgG2: IgG2 deposited in the glomeruli; tIgG3: IgG3 deposited in the glomeruli; tIgG4:IgG4 deposited in the glomeruli.

Immunofluorescence revealed IgG4 deposits in all patients. Additionally, IgG1 deposits were present in 112 patients (96.55%), IgG3 in 89 (76.72%), and IgG2 in 62 (53.45%). The mean intensity of IgG1, IgG2, IgG3 and IgG4 in all cohort were 1.66, 0.68, 1.13, 2.32, respectively. The positive rate and immunostaining intensity of IgG subclasses and complement compartment deposited in the glomeruli were not different between the early- and advanced- stages (Table 2). The correlation between the IF intensities of IgG subclasses and complement activation products (C3 and C1q) was also examined. The IF intensity of IgG1 (*r* = 0.15, *p* = .01) and IgG3 (*r* = 0.17, *p* = .005) was positive correlative with the intensity of C3. Moreover, the IgG1 (*r* = 0.18, *p* = .002) and the IgG3 (*r* = 0.27, *p* < .001) were also positive correlative with C1q, too. No significant relation was found between IgG2 and IgG4 with C3 and C1q (Figure 2).

**Table 2.** The IgG subclasses and complement distribution in early and advanced EM stages.

	Total (*n* = 116)	I + II (*n* = 92)	III+IV (*n* = 24)	*p*
IgG1 positive, *n* (%)	112 (96.55)	89 (96.74)	23 (95.83)	1.000
IgG1 intensity, mean ± SD	1.66 ± 0.65	1.65 ± 0.65	1.67 ± 0.64	0.923
IgG2 positive, *n* (%)	62 (53.45)	52 (56.52)	10 (41.67)	0.194
IgG2 intensity, mean ± SD	0.68 ± 0.72	0.72 ± 0.72	0.54 ± 0.72	0.287
IgG3 positive, *n* (%)	89 (76.72)	72 (78.26)	17 (70.83)	0.443
IgG3 intensity, mean ± SD	1.13 ± 0.81	1.13 ± 0.80	1.12 ± 0.85	0.977
IgG4 positive, *n* (%)	116 (100)	92 (100)	24 (100)	–
IgG4 intensity, mean ± SD	2.32 ± 0.58	2.32 ± 0.57	2.33 ± 0.64	0.893
C3 positive, *n* (%)	107 (92.24)	83 (90.22)	24 (100.00)	0.243
C3 intensity, mean ± SD	1.62 ± 0.77	1.58 ± 0.79	1.79 ± 0.66	0.220
C1q positive, *n* (%)	21 (18.10)	18 (19.57)	3 (12.50)	0.615
C1q intensity, mean ± SD	0.22 ± 0.49	0.23 ± 0.49	0.17 ± 0.48	0.586

### Correlation analysis of serum PLA2R-IgG4/PLA2R-IgG with the clinical parameters

**Figure 3.**
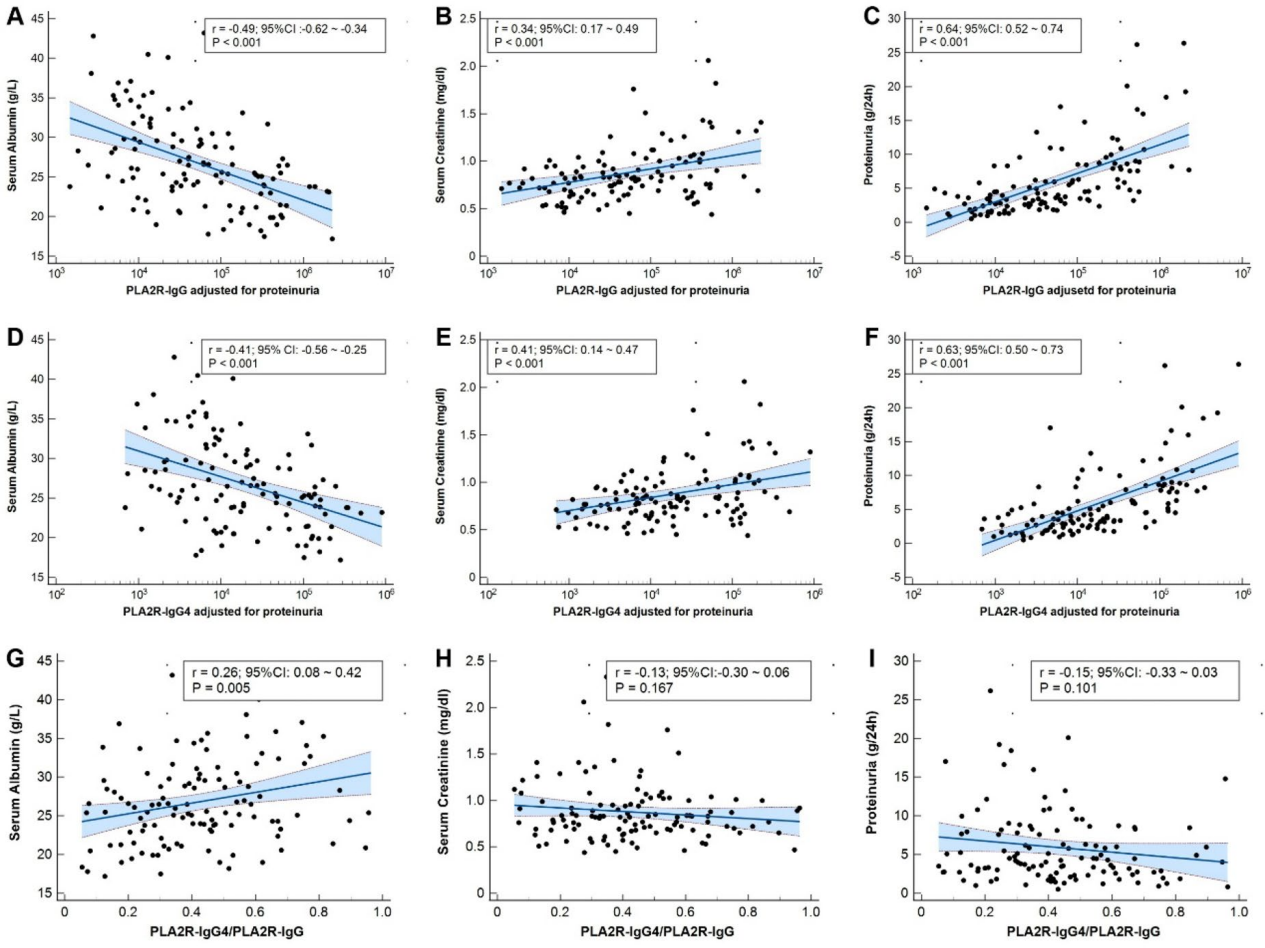
The correlation of adjusted PLA2R-IgG level with serum albumin (A), serum creatinine (B) and proteinuria (C). The correlation of adjusted PLA2R-IgG4 level with serum albumin(D), serum creatinine (E) and proteinuria (F). The correlation of PLA2R-IgG4/PLA2R-IgG with serum albumin (G), serum creatinine (H) and proteinuria (I).

The level of PLA2R-IgG4 were strong positively correlated with PLA2R-IgG (*r* = 0.852, *p* < .001). Given that urinary immunoglobulin loss is a significant confounding factor in the accurate estimation of serum antibody levels, we therefore adjusted the measured levels of PLA2R-IgG and PLA2R-IgG4 with 24-h proteinuria. The adjusted level of PLA2R-IgG was positively correlated with 24-hour proteinuria (*r* = 0.64, *p* < .001) and serum creatinine (*r* = 0.34, *p* < .001), negatively correlated with serum albumin (*r* = −0.49, *p* < .001) (Figure 3A–C). Meanwhile, the adjusted level of PLA2R-IgG4 was also positively correlated with 24-h proteinuria (*r* = 0.63, *p* < .001) and serum creatinine (*r* = 0.41, *p* < .001), negatively correlated with serum albumin (*r* = −0.41, *p* < .001) (Figure 3D-F). On the contrary, the PLA2R-IgG4/PLA2R-IgG was significantly positively correlated with serum albumin (*r* = 0.26, *p* = .005), negatively with serum creatinine (*r* = −0.13, *p* = .17) and proteinuria (*r* = −0.15, *p* = .10) but without statistically significant (Figure 3G–I). It is noticed that the PLA2R-IgG4/PLA2R-IgG ratio demonstrated a significant negative correlation with the adjusted PLA2R-IgG level (*r* = −0.37, *p* < .001), whereas no significant correlation was found with the adjusted PLA2R-IgG4 level.

### Correlation analysis between PLA2R-IgG4/PLA2R-IgG with the intensity of IgG subclass and complement

**Figure 4.**
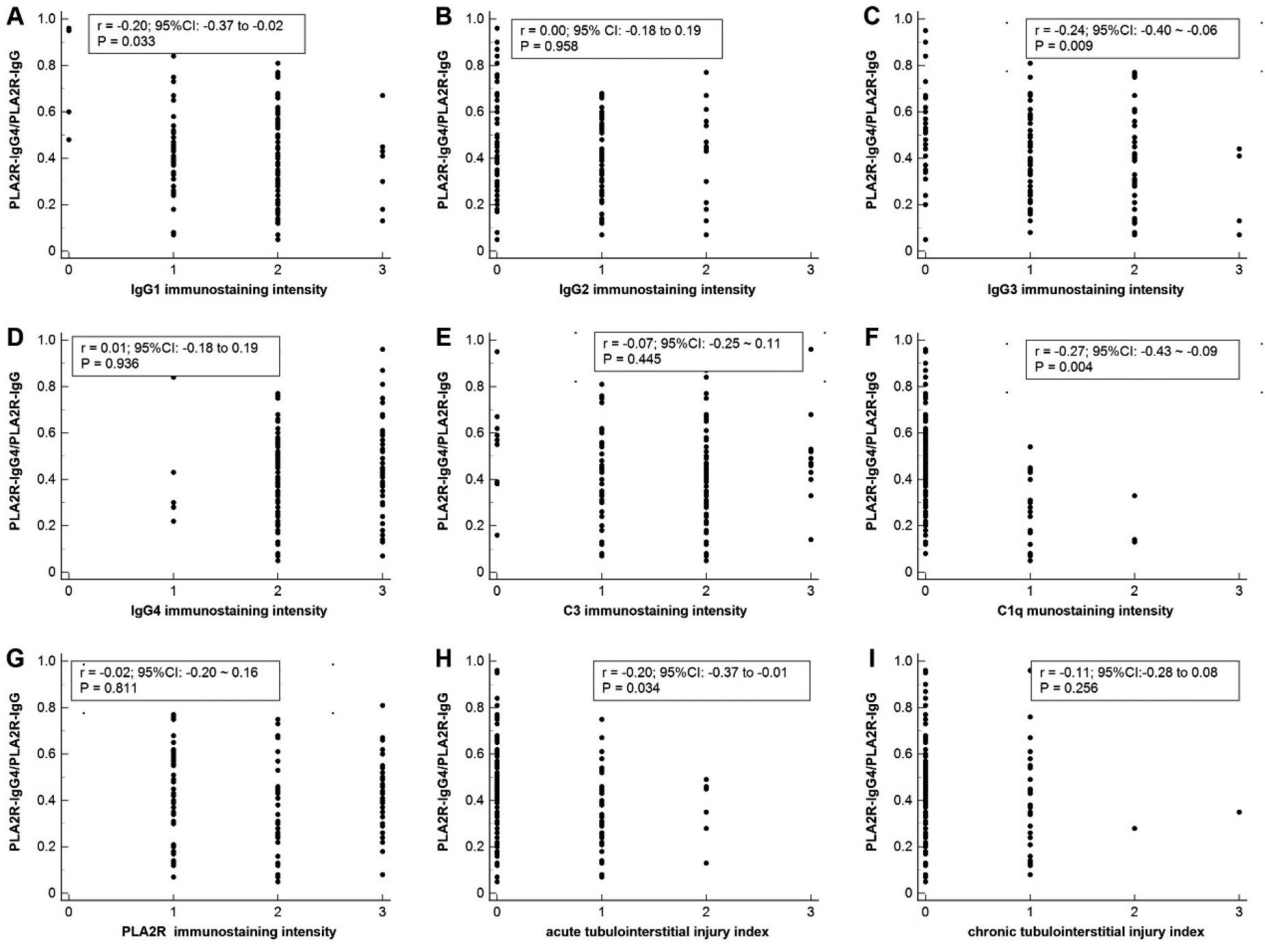
The correlation of PLA2R-IgG4/PLA2R-IgG with the intensity of IgG subclass, PLA2R, C3, C1q, acute tubulointerstitial injury index and chronic tubulointerstitial injury index. PLA2R: phospholipase A2 receptor.

PLA2R-IgG4/PLA2R-IgG is negatively correlated with the intensity of IgG1 and IgG3 deposited in the glomeruli (*r* = −0.20, *p* = .03; *r* = −0.24, *p* = .009) (Figure 4A and C), but no correlation was found between PLA2R-IgG4/PLA2R-IgG with the intensity of IgG2 and IgG4 (Figure 4B and D). There was no correlation between PLA2R-IgG4/PLA2R-IgG and the intensity of C3 (Figure 4E). But the PLA2R-IgG4/PLA2R-IgG were negatively correlated with the intensity of C1q staining (*r* = −0.27, *p* = .004) (Figure 4F), as the PLA2R-IgG4/PLA2R-IgG increase, the intensity of C1q were decease. We also examined the relationship between PLA2R staining intensity and the serum PLA2R-IgG4/PLA2R-IgG ratio, but no significant correlation was observed (Figure 4G). The PLA2R-IgG4/PLA2R-IgG also negatively correlated with the acute tubulointerstitial injury index (*r* = −0.20, *p* = .03), but no correlation with the chronic tubulointerstitial injury index (Figure 4H and I).

### The relationship between PLA2R-IgG4/PLA2R-IgG with EM stages

**Figure 5.**
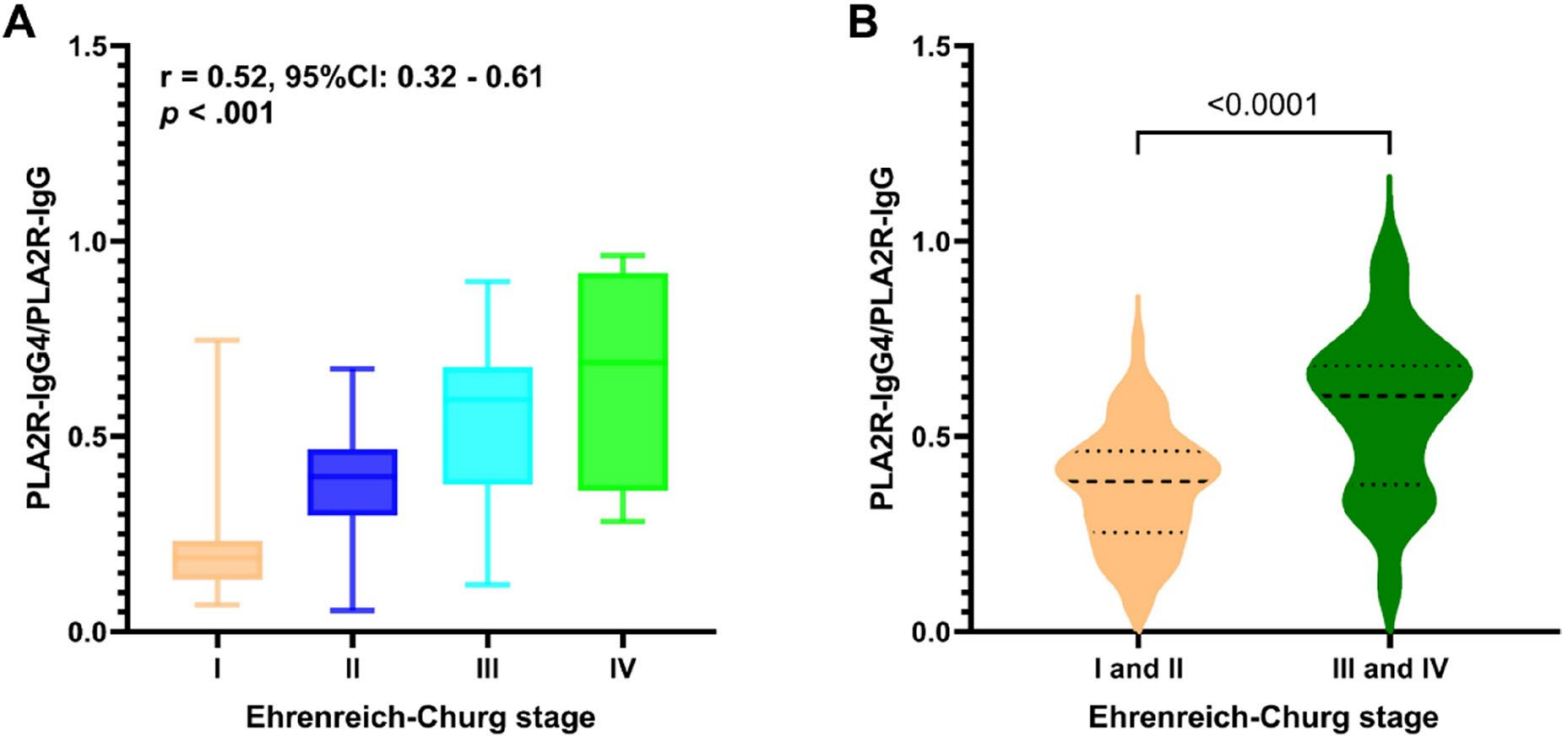
The relationship between PLA2R-IgG4/PLA2R-IgG with Ehrenreich–Churg stages.

EM stages of MN were predominantly distributed in Stage II and III among the study patients, accounting for 86.21% (100/116) of cases. Stage I and IV disease were less common, only comprising 13.79% (16/116). The median PLA2R-IgG4/PLA2R-IgG ratio was 18.92% in Stage I, 39.74% in Stage II, 59.38% in Stage III, and 68.99% in Stage IV (Table 3). PLA2R-IgG4/PLA2R-IgG was significantly positively correlated with EM stages (*r* = 0.52, 95% CI: 0.32–0.61, *p* < .001; Figure 5A). The PLA2R-IgG4/PLA2R-IgG ratio was significantly higher in advanced stages (III + IV) compared to early stages (I + II) (Figure 5B). To determine if this ratio remained independently associated with the EM stages, we performed a multivariate logistic regression analysis that included age, gender, disease course, serum albumin, and serum creatinine. The results demonstrated that both serum creatinine (OR: 10.35, 95% CI: 1.82–58.91, *p* = .01) and the PLA2R-IgG4/PLA2R-IgG ratio (OR: 1951.94, 95% CI: 15.48–246141.75, *p* = .002) were independent predictors of advanced EM stages (Table 4).

**Table 3.** The correlation of PLA2R-IgG4/PLA2R-IgG with the EM stages.

Stage	I	II	III	IV	*p*
*N*	12	80	20	4	
PLA2R-IgG4/PLA2R-IgG, %	18.92 (13.40–23.20)	39.74 (29.74–46.68)	59.38 (37.61–67.74)	68.99 (36.06–91.86)	0.008

**Table 4.** Correlations between EM stages and clinical features in PLA2R associated patients: univariate and multivariate logistic regression analysis.

	Univariable analysis		Multivariate analysis	
	OR (95% CI)	*p*	OR (95% CI)	*p*
Male	1.23 (0.46–3.29)	0.68		
Age	0.97 (0.93–1.01)	0.14	0.99 (0.94–1.03)	0.56
Course	1.02 (0.97–1.08)	0.35		
Albumin	1.02 (0.94–1.10)	0.61		
Creatinine	3.42 (0.92–12.68)	0.07	10.35 (1.82–58.91)	**0.01**
PLA2R-IgG	1.00 (1.00–1.00)	0.1	1.00 (1.00–1.00)	0.4
Ratio	1745.01 (55.44–54925.70)	**<.001**	2280.00 (44.36–117191.52)	**<.001**

OR: odds ratio; CI: confidence interval; Ratio: PLA2R-IgG4/PLA2R-IgG.

### The clinicopathological characters of different PLA2R-IgG4/PLA2R-IgG groups

Patients were stratified into four groups based on the median PLA2R-IgG4/PLA2R-IgG ratio. As this ratio increased, serum albumin levels significantly increased (*p* < .01), while PLA2R-IgG levels significantly decreased (*p* < .01). Concurrently, the intensity of glomerular C1q deposition also decreased. Furthermore, advanced EM stages were observed more frequently with higher PLA2R-IgG4/PLA2R-IgG ratios (Table 5).

**Table 5.** The clinicopathological characters of different PLA2R-IgG4/PLA2R-IgG subgroups.

Variables	Quartiles PLA2R-IgG4/PLA2R-IgG ratio				*p*
	< 0.28 (*n* = 29)	0.28≤*x* < 0.40 (*n* = 29)	0.40≤*x* < 0.52 (*n* = 29)	>0.52 (*n* = 29)	
Cr (mg/dl)	0.79 (0.72, 1.12)	0.82 (0.61,0.90)	0.83 (0.71,1.00)	0.83 (0.69,1.00)	0.73
Alb (g/l)	23.8 (20.5, 26.6)	26.5 (22.1,29.8)	26.6 (23.9,29.8)	27.0 (24.3,33.1)	0.04
UPro (g/24 h)	4.53 (2.84, 9.03)	4.21 (3.46,7.84)	4.90 (2.19,9.57)	3.38 (2.43,5.85)	0.27
PLA2R-IgG (ng/ml)	25946.73 (12348.75, 56538.05)	15246.50 (5163.33,43549.56)	10004.53 (4296.73,23445.22)	8696.30 (2938.59,11147.90)	<.01
PLA2R-IgG4 (ng/ml)	4385.89 (1654.56, 15427.89)	6152.16 (2043.43,13769.10)	4271.04 (1385.74,11350.87)	5420.17 (1947.78,7787.82)	0.80
IgG, *n* (%)					0.96
2	11 (37.93)	9 (31.03)	10 (34.48)	10 (34.48)	
3	18 (62.07)	20 (68.97)	19 (65.52)	19 (65.52)	
C3, *n* (%)					0.17
0	1 (3.45)	3 (10.34)	0 (0.00)	5 (17.24)	
1	10 (34.48)	9 (31.03)	7 (24.14)	11 (37.93)	
2	17 (58.62)	15 (51.72)	17 (58.62)	10 (34.48)	
3	1 (3.45)	2 (6.90)	5 (17.24)	3 (10.34)	
C1q, *n* (%)					<.01
0	18 (62.07)	25 (86.21)	24 (82.76)	28 (96.55)	
1	9 (31.03)	2 (6.90)	5 (17.24)	1 (3.45)	
2	2 (6.90)	2 (6.90)	0 (0.00)	0 (0.00)	
PLA2R, *n* (%)					0.13
1	9 (31.03)	12 (41.38)	9 (31.03)	12 (41.38)	
2	16 (55.17)	7 (24.14)	8 (27.59)	9 (31.03)	
3	4 (13.79)	10 (34.48)	12 (41.38)	8 (27.59)	
EM, *n* (%)					<.01
I + II	27 (93.10)	25 (86.21)	27 (93.10)	13 (44.83)	
III + IV	2 (6.90)	4 (13.79)	2 (6.90)	16 (55.17)	

Cr: serum creatinine; Alb: serum albumin; Upro: 24-h urine protein; IgG: immunoglobulin G immunofluorescence intensity deposited in glomeruli; C3: complement C3 immunofluorescence intensity deposited in glomeruli; C1q: complement C1q immunofluorescence intensity deposited in glomeruli; PLA2R: phospholipase A2 receptor immunofluorescence intensity deposited in glomeruli; EM: electron microscopy Ehrenreich–Churg stage.

## Discussion

Our preliminary observations in this study revealed an increasing trend of PLA2R-IgG4/PLA2R-IgG ratio from stage I to stage IV in PLA2R-associated MN. The PLA2R-IgG4/PLA2R-IgG ratio is an independent predictor of advanced EM stages. Meanwhile, as the PLA2R-IgG4/PLA2R-IgG increase, the intensity of IgG1, IgG3 and C1q deposited in glomeruli decreased. The observed change in IgG subclass predominance from early to advanced EM stages is consistent with the hypothesis of an autoantibody subclass switch during disease progression. It is possible that the IgG1 and IgG3 anti-PLA2R response dominant in early stages, which, with disease progression, will turn into an IgG4-dominant antibody response. And the classical complement activation pathway may contribute the membranous attack complex formation, as the IgG1/IgG3 and C1q is often founded in the early stage of MN. Our results further advance the understanding of pathogenic PLA2R autoantibodies, demonstrating that, not only do the levels of total anti-PLA2R antibody fluctuate, but the proportions of its IgG subclasses also exhibit different among different EM stages. However, as this is a cross-sectional study, this mechanistic inference requires validation through longitudinal studies.

Immunofluorescence staining for IgG subclasses serves as a critical diagnostic tool for distinguishing PMN from secondary MN (SMN). Predominant glomerular IgG4 deposition is highly suggestive of PMN, while a predominance of IgG1 or IgG3 should raise suspicion for SMN. However, IgG1/IgG3-dominant or codominant staining patterns warrant cautious interpretation in diagnosing SMN without obvious underlying disease, as IgG1 or IgG3 predominance may also be present in early-stage PMN [13]. Concurrently, C1q deposition-traditionally considered indicative of SMN – also occurs with significant frequency in early-stage PMN, suggesting prominent involvement of the classical complement pathway during initial disease phases [20,21]. Our findings further revealed that IgG3 staining intensity positively correlated with C3 and C1q deposition, which indicating that IgG3 may initiate classical pathway activation *via* C1q binding in early disease. Furthermore, an inverse relationship was observed between the serum PLA2R-IgG4/PLA2R-IgG ratio and glomerular IgG1, IgG3 and C1q immunostaining intensity, supporting a potential antagonistic role of IgG4 against IgG3-mediated classical pathway activation. These findings support the following hypothesis: IgG1 and/or IgG3 subclass of anti-PLA2R antibodies bind antigen within glomeruli, activating the classical complement cascade and leading to C1q deposition, to forming the immune complex in early stage. During disease progression, an IgG subclass switch to IgG4 predominance occurs, potentially shifting complement activation toward the mannose-binding lectin (MBL) or alternative pathways in the later stages. This may part explain the complement activation pathway in different observation studies because using the different stages patient samples, as some researchers believe the MBL is the predominant complement pathway [22–25], while others support the classical complement pathway plays an important role [20,26].

Immunoglobulin subclass switching dynamically correlates with disease progression across autoimmune disorders. In bullous pemphigoid, transition from pro-inflammatory IgG1 to functionally blocking IgG4 correlates with clinical remission [27]. Analogously, autoimmune nodopathy mediated by contactin-1 (CNTN1) antibodies features transient IgG1 autoantibodies during acute flares that become undetectable upon stabilization, positioning IgG1 as an activity biomarker [28]. Despite shared genetic etiology in anti-neutral endopeptidase (NEP) antibody-mediated MN, divergent maternal IgG subclass profiles critically modulate disease severity through complement-driven cytotoxicity versus enzyme-inhibitory mechanisms [29]. Significantly, IgG3 anti-NEP subclass portends poor renal outcomes, whereas IgG4 predominance associates with favorable prognosis [30], underscoring subclass-specific pathobiological impacts.

The results about the IgG subclasses switch in PLA2R-associated MN are limited and controversial. Most PLA2R-associated MN patients present with significant proteinuria at the time of diagnosis, indicating a more advanced disease stage, biopsies at this point predominantly show IgG4 deposits. Moreover, it is hard to trajectory the IgG subclass switch during the disease progress through kidney biopsies because it’s an invasive method. Evidence suggests variability in IgG subclass dominance across different stages of PMN. IgG1-dominant or codominant staining is observed in a majority of early-stage cases [31]. In contrast, IgG4-dominant or codominant deposits predominate (>80%) in later stages, implying a potential shift from an early IgG1-predominant response toward IgG4 as disease evolves [13,31]. However, further studies describe persistent IgG4-dominance across all stages without significant variation in prevalence [32] and report no correlation between IgG4 intensity and EM stages [33]. Analysis of a cohort similarly found no statistical evidence of subclass switching from IgG1 to IgG4 with advancing EM stage; however, IgG3 deposition intensity declined significantly with stage progression [14]. In renal transplants protocol biopsies, recurrent MN commonly exhibits a stable IgG dominance pattern over time, potentially reflecting the persistence of pre-formed, subclass-switched anti-PLA2R antibodies from the native disease prior to transplantation [15].

The discrepancy results drawn from the above studies may be partially attributed to the intensity of IF being subjectively reliant on the pathological experience or may be influenced by the procession of renal biopsy tissues. Muchmore, the pathogenic antibodies depositing in renal tissue and binding to target antigens represent only a minor fraction of the systemic autoantibody pool, particularly during active disease phases characterized by high serum levels of anti-PLA2R antibody. This is consistent with the theoretical ‘kidney sink’ hypothesis, which posits that renal antigen binding epitope can become saturated, leading to a plateau phase in antibody deposition. Consequently, the serum PLA2R-IgG4/PLA2R-IgG ratio may provide a more direct assessment of its correlation with the stages of PMN. To our knowledge, this study is the first to specifically investigate the relationship between the serum PLA2R-IgG4/PLA2R-IgG ratio and EM stages in PMN. A key finding is the significant rise in this ratio in advanced stages, which provides compelling evidence that the autoantibody subclasses differ across EM stages, indicative of an IgG subclass switch favoring PLA2R-IgG4 as the disease advances.

Although our study has observed the difference of subclass predominance among different EM stages, the mechanistic details which drive this difference and the meaningful of subclass switch toward to IgG4 subclass warrant further elucidation. Crucially, IgG1 and IgG3 are potent effectors capable of activating the complement cascade and mediating antibody-dependent cellular cytotoxicity (ADCC) *via* Fcγ receptor engagement, mechanisms directly associated with severe inflammatory tissue injury. Conversely, IgG4 possesses limited complement-activating potential and exhibits unique properties such as Fab-arm exchange that result in functionally monovalent/bispecific antibodies, generally associated with anti-inflammatory effects. This predominance of IgG4 likely contributes to the characteristic paucity of inflammatory cell infiltration observed in most PMN biopsies; when present, significant inflammation may correlate with poorer prognostic outcomes [34,35]. Notably, emerging evidence suggests that altered glycosylation patterns in IgG4 may promote podocyte injury *via* activation of the lectin complement pathway [24]. Therefore, critical unanswered questions persist: why and how a preferential class switch towards IgG4-an isotype often considered immunologically ‘protective’-occurs in PMN patients, and whether this represents the cause or consequence of disease chronicity. The strong association between IgG4 dominance within the renal deposits and serum autoantibody profile suggests it marks a later stage in the autoimmune response trajectory [36,37]. Clinically, an increasing PLA2R-IgG4/PLA2R-IgG ratio accompanied by declining total anti-PLA2R antibody levels may serve as a serologic indicator of a shift towards immunological quiescence and favorable prognosis. It is noteworthy that a newly research found that the anti-PLA2R IgG4-to-IgG ratio helps predict remission of PLA2R-associated MN. High ratio seems have a good outcome [38]. This immunomodulatory phenomenon potentially explains the approximately 30% spontaneous remission rate observed in PMN patients.

Despite the intriguing findings presented here, this study has several limitations that warrant acknowledgment. First, this is an observational and cross-sectional investigation. Personal ratio changes during follow-up to confirm the ratio change and impact of the ratio change on the prognosis need further study. Second, the sample sizes of patients with stage I and stage IV disease were relatively small. Therefore, the findings pertaining to these subgroups warrant cautious interpretation. Future studies including larger cohorts of patients representing earlier (stage I) and more advanced (stage IV) stages are needed to validate our findings. Third, the potential prognostic significance of varying baseline ratios remains unclear; further longitudinal follow-up is required to determine whether differential baseline ratios are associated with distinct long-term outcomes.

## Conclusions

Collectively, by integrating the assessment of the serum PLA2R-IgG4/PLA2R-IgG ratio with direct analysis of glomerular-deposited IgG subclasses, our investigation reveals distinct anti-PLA2R antibody subclass profiles across different histopathological stages. The results highlight a heterogeneous immune response across disease stages. This heterogeneity suggests involvement of the classical complement pathway in mediating podocyte injury during the early stages of MN. However, the subsequent predominance of PLA2R-IgG4 implies a potential shift later in the disease course, where the MBL pathway may subsequently assume a primary role in complement activation. These findings underscore the necessity for further research into the distinct pathogenic contributions of specific IgG subclasses in MN.

## Data Availability

The datasets used and/or analyzed during the current study are available from the corresponding author on reasonable request.
